# Mesenchymal stem cell-derived extracellular vesicles prevent the development of osteoarthritis via the circHIPK3/miR-124-3p/MYH9 axis

**DOI:** 10.1186/s12951-021-00940-2

**Published:** 2021-06-30

**Authors:** Shenglong Li, Jie Liu, Siyu Liu, Weijie Jiao, Xiaohong Wang

**Affiliations:** 1 Department of Tissue Engineering, Center of 3D Printing & Organ Manufacturing, School of Fundamental Sciences, China Medical University (CMU), No. 77 Puhe Road, Shenyang North New Area, Shenyang, 110122 China; 2 Department of Bone and Soft Tissue Tumor Surgery, Cancer Hospital of China Medical University, Liaoning Cancer Hospital & Institute, Shenyang, 110042 Liaoning Province China; 3 Department of Prosthodontics, School and Hospital of Stomatology, China Medical University, Liaoning Provincial Key Laboratory of Oral Diseases, Shenyang, 110002 China; 4 Center of Organ Manufacturing, Department of Mechanical Engineering, Tsinghua University, Beijing, 100084 China

**Keywords:** Mesenchymal stem cells (MSCs), Extracellular vesicles, Osteoarthritis, Circular RNA HIPK3 (circHIPK3), MiR-124-3p, MYH9

## Abstract

**Background:**

Extracellular vesicles (EVs) secreted by mesenchymal stem cells (MSCs) may play a vital role in a variety of biological processes, including cartilage regeneration. However, few studies reported their potential in the development of osteoarthritis (OA) previously. In this study, we explored the biological roles and underlying mechanism of MSCs-EVs in OA.

**Results:**

Co-culture experiments revealed that MSCs-EVs could promote the expression of collagen type II alpha 1 chain (COL2A1), SRY-box transcription factor 9 (SOX9) and Aggrecan while negatively regulate the expression of chondrocyte hypertrophy markers matrix metallopeptidase 13 (MMP-13) and RUNX family transcription factor 2 (Runx2) in mouse chondrocytes in the OA model. Besides, the results of cell experiments indicated that MSCs-EVs could notably weaken the suppression of chondrocyte proliferation, migration and the promotion of chondrocyte apoptosis via interleukin1β (IL-1β) induction. In addition, MSCs-circHIPK3-EVs (EVs derived from MSCs overexpressing circHIPK3) considerably improved IL-1β-induced chondrocyte injury. Mechanistically, we elucidated that circHIPK3 could directly bind to miR-124-3p and subsequently elevate the expression of the target gene MYH9.

**Conclusion:**

The findings in our study demonstrated that EVs-circHIPK3 participated in MSCs-EVs-mediated chondrocyte proliferation and migration induction and in chondrocyte apoptosis inhibition via the miR-124-3p/MYH9 axis. This offers a promising novel cell-free therapy for treating OA.

**Graphic abstract:**

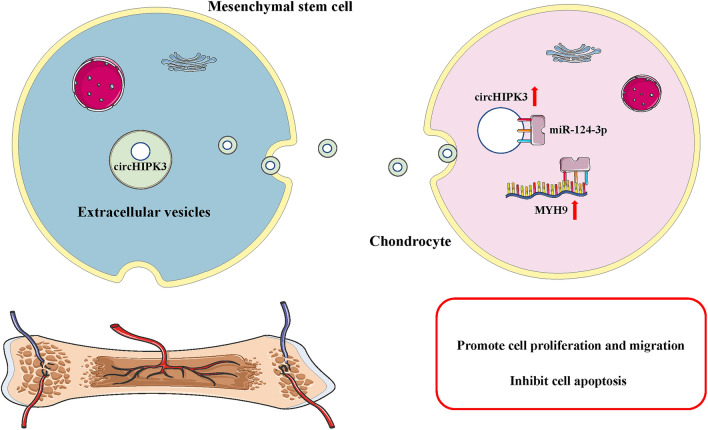

## Background

Osteoarthritis (OA) is a common degenerative disorder of the joints that accounts for major physical pain in older adults [1, 2]. OA could result in severe joint pain, stiffness, limited motion, disability, and in serious cases, the loss of joint mobility [3, 4]. OA is regarded as the leading cause of lower limb disability, with a disability rate of up to 53% [5, 6]. Studies indicate that the incidence of OA increases annually worldwide [7], which not only has a strong impact on the labor ability and quality of life of patients, but also brings a huge economic burden to society. However, current medical interventions for OA have led to poor clinical outcomes, demonstrating that there are huge unmet medical needs in this area. The occurrence and development of OA are associated with complex interactions among many factors, such as mechanical, cellular, and biochemical factors [8], while the pathogenesis of OA remains unclear. Hence, exploring the novel therapeutic approach for OA is critical.

Mesenchymal stem cells (MSCs), a group of multi-potential stem cells exhibit self-renewal and tissue differentiation ability, are capable of differentiating into endodermal, mesodermal, ectodermal, and other cell populations in vivo and in vitro [9, 10]. In addition to bone marrow, MSCs are also derived from fat, umbilical cord blood, peripheral blood, placenta, skin, amniotic fluid, synovial membrane, teeth root, and other tissues [11, 12]. MSCs are easy to culture and proliferate in vitro, have strong properties of anti-inflammatory and immunomodulatory, and play a vital role in the repair and regeneration of various tissues [13, 14]. Recently, MSCs have become the most promising seed cell for cartilage repair due to their wide range of sources, less trauma, strong proliferation, and good cartilage differentiation potentials, and have been widely researched and applied [15]. Many studies demonstrate that MSCs from bone marrow, fat and other sources have been applied to clinical treatment of cardiovascular diseases, nervous system diseases, immune diseases, bone and joint diseases [16–22]. Recently, there has been a notable paradigm shift in the mechanism of action of MSCs in tissue repair. Studies have shown that under specific induction conditions, MSCs cultured in vitro can differentiate into chondrocytes, and the formation of cartilage mimics the development and growth of embryonic cartilage [23]. Different cytokines and growth factors from MSCs, such as insulin-like growth factor (IGF), bone morphogenetic protein (BMP), and transfer growth factor β (TGF-β), have the ability to promote the repair of cartilage tissue [24]. Besides, the anti-inflammatory factors secreted by MSCs and their inhibtion effects on immune cell proliferation play a major role in the repair of OA inflammation [25–29].

Nowdays, more and more studies demonstrate that Nanomaterials play an important role in the process of gene delivery and information exchange [30, 31]. It is increasingly evident that the therapeutic effects of MSCs are largely attributed to their paracrine secretion. The secreted factors, collectively known as the secretome (or secretomes), are composed of soluble proteins, free nucleic acids, lipids, and extracellular vesicles (EVs) [9, 32]. On the basis of biogenesis and size, EVs can be divided into three main types: exosomes (30–150 nm in diameter), microvesicles/microparticles, and apoptotic bodies (both considered to be > 100 nm) [33, 34]. Exosomes are secreted to the extracellular environment through the fusion of multivesicular bodies with the plasma membrane. While microvesicles and apoptotic bodies are released through forward budding of the plasma membrane in living and dying cells, respectively [35, 36]. The crucial role of MSCs-derived EVs for the regulation of cell migration, proliferation, differentiation, and extracellular matrix (ECM) synthesis has been increasingly supported by recent findings [37, 38]. Zhang and Cosenza et al. proved that intra-articular injection of MSCs-EVs can restore cartilage and subchondral bone in the defect [39, 40]. Compared with the cell therapy of MSCs, MSC-EVs represents a safer and more effective treatment way.

Data from the Human Genome Project demonstrates that genes with coding functions account for only approximately 1% of the total genome sequence, and most of the remaining transcription products are non-coding RNAs (ncRNAs), which do not have the capability of protein-coding [41]. In recent years, ncRNAs have become a research hotspot in the field of molecular biology. Together with the more mature miRNAs, it has been proven that ncRNAs have great potential to regulate gene expression and been clinical biomarkers or therapeutic targets [42]. Circular RNAs (circRNAs) are a novel class of discovered ncRNAs with structural stability, formed by reverse splicing of the 3′-end and 5′-end of a chain-like pre-mRNA [43, 44]. In general, circRNAs are formed via exon or intron cyclization, has no 3′-end or 5′-end of linear RNA, their structure is very stable and cannot be hydrolyzed by ordinary RNase; [45–47]. Circular RNA homeodomain-interacting protein kinase three (circHIPK3) is produced from exon two of HIPK3 and have been studied in many recent studies. HIPK3 (GenBank Accession ID NM_005734.5) is located on chromosome 11p13 and includes 7,551 base pairs [48, 49]. The genome sequence revealed that the second exon (1,099 bp) of HIPK3, as well as the long introns at both ends of the gene, combine to form the structure of circHIPK3 [49]. CircHIPK3 has been proven to play a vital regulatory role in many diseases such as lung cancer [49], pulmonary fibrosis [50], hepatocellular carcinoma [51] and bladder cancer [52]. However, its role in OA requires further exploration. More importantly, circHIPK3 has been confirmed to be more abundant in EVs [53], this means that circHIPK3 may play an important role through cell-to-cell communication. Besides, circHIPK3 has also been reported to having the ability of promoting cell proliferation and regeneration [54]. Overexpression of circHIPK3 may reduce the oxidative damage of human osteoblasts by hydrogen peroxide and suppress cell apoptosis [55]. These studies indicate that circHIPK3 may play an important regulatory role in OA.

In this study, we found that circHIPK3, which is derived from MSCs-EVs, remarkedly promote chondrocyte proliferation, migration and suppress apoptosis. Mechanistically, circHIPK3 may act as an endogenous competitive RNA to promote MYH9 expression by binding to miR-124-3p. Therefore, we hypothesized that EVs-circHIPK3 may play a vital role in both chondrogenic proliferation and OA pathogenesis.

## Results

### Isolation and characterisation of MSC-derived EVs

**Fig. 1:**
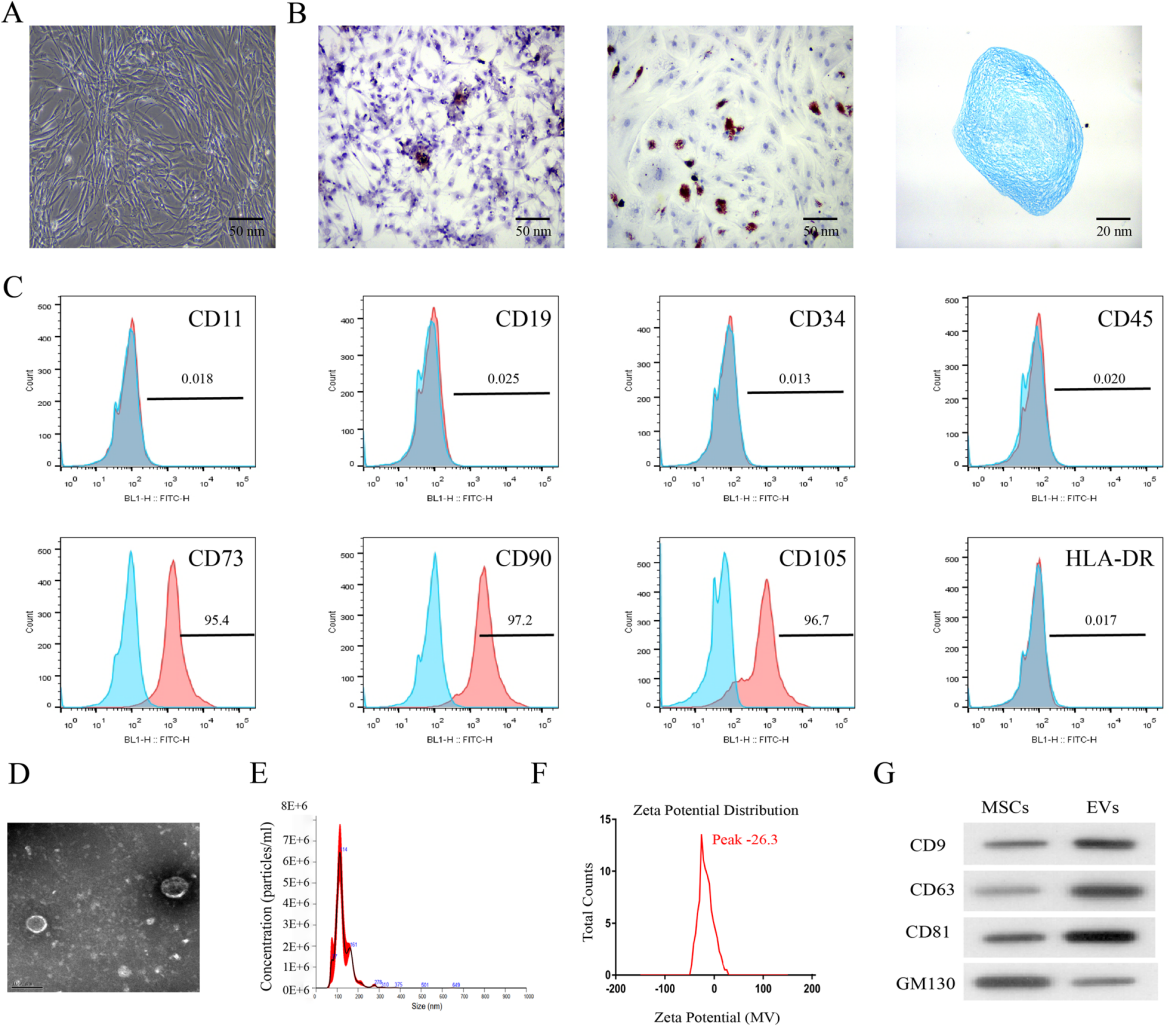
Isolation and characterisation of MSC-derived EVs. **A** After the culture of mesenchymal stem cells (MSCs), microscopic observation showed a typical spindle shape of cells. **B** Extracted MSCs had osteogenic, adipogenic and chondrogenic potentials by Alizarin Red, Oil Red O and Alcian Blue staining. **C** MSC positive markers (CD73, CD90 and CD105) and negative markers (HLA-DR, CD11b, CD19, CD34 and CD45) were analyzed by flow cytometry. **D** The morphology of EVs was observed under a transmission electron microscope. **E** The particle size distribution of MSC derived EVs was measured by nanometer size analyzer. **F** Zeta potential measurements of the surface charge of MSCs-EVs (mV). **G** EVs markers (CD9, CD63, CD81 and GM130) were analyzed by Western blot

MSCs were identified at passage three for subsequent experiments. After in vitro culture, microscopic examination showed a relatively uniform spindle-shaped cell population and a significantly higher proliferation of MSCs (Fig. 1A). MSCs were cultured in different media to test the abilities of differentiation. The results of Alizarin Red, Oil Red O, and Alcian Blue staining showed that the extracted MSCs had the potential to differentiate into osteogenic, adipogenic, and chondrogenic cells (Fig. 1B). Then, we conducted flow cytometer assay to detect the surface markers of stem cells, which indicated that the extracted MSCs expressed CD73, CD90, and CD105, and could not express CD11b, CD19, CD34, CD45, and HLA-DR (Fig. 1C). These results verified that the extracted MSCs were pure. Furthermore, we extracted EVs from MSCs using ultracentrifugation and adopted transmission electron microscopy (TEM), NanoSight, zeta potential and western blot assay to evaluate the isolated EVs. The TEM results showed that MSCs-EVs presented a bilayer lipid structure with a diameter of 50–150 nm and a significant cup-shaped depression in the middle (Fig. 1D). The nanometer size analysis showed that the diameter of most of the MSCs-EVs was approximately 50 -150 nm (Fig. 1E). The surface charge of MSCs-EVs was also quantified. MSCs-EVs presented a negative surface charge, as determined through zeta potential measurements (Fig. 1F). The peak of the zeta potential was about -26.3 mV, which meant MSCs-EVs might be stable. Besides, immunoblotting results revealed that the MSCs-EVs expressed EVs markers, such as CD9, CD63, and CD81, and were associated with a dramatically lower expression of GM130 (Fig. 1G).

### Influence of EVs-circHIPK3 on chondrocyte proliferation, migration, and apoptosis

**Fig. 2:**
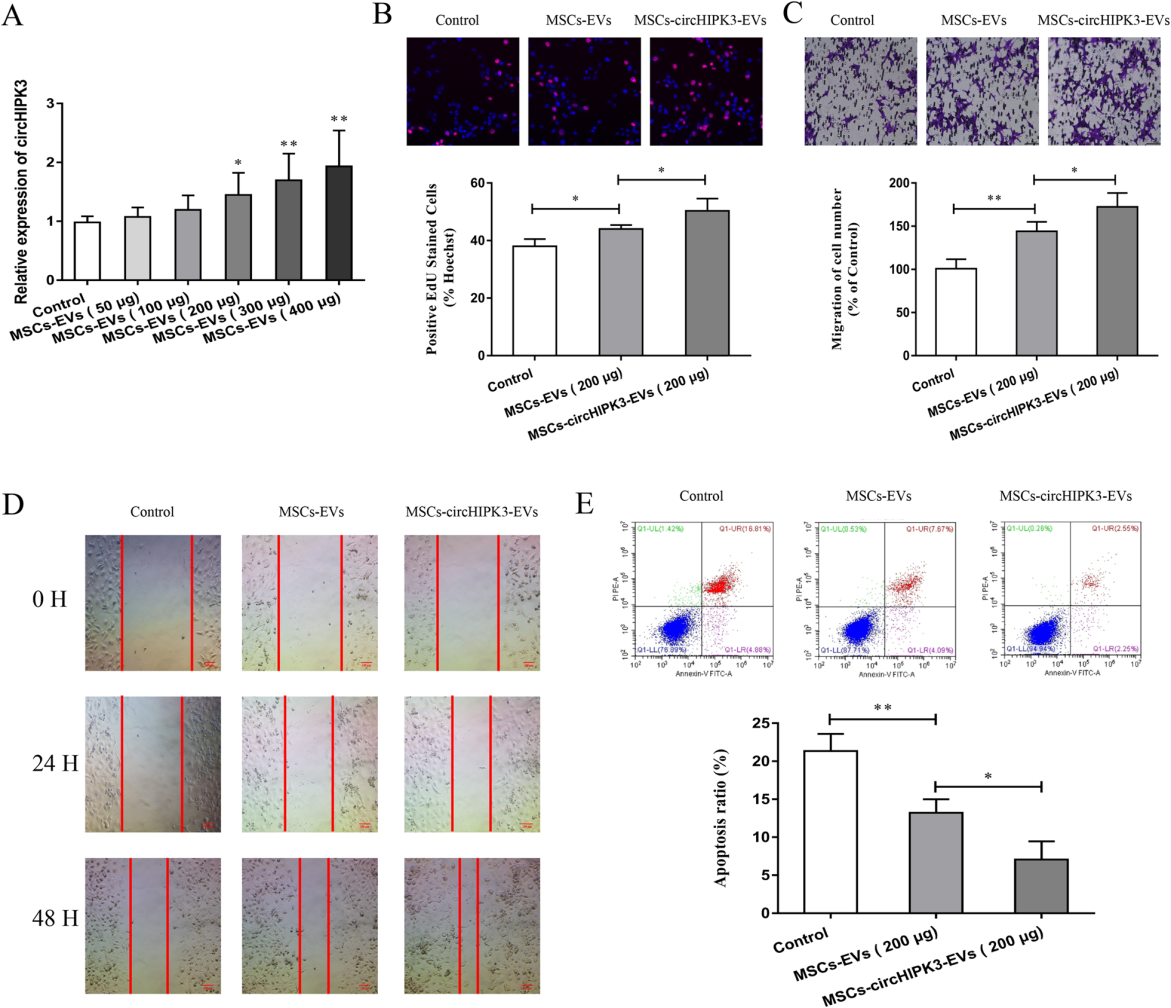
Influence of EVs-circHIPK3 on the proliferation, migration and apoptosis of chondrocytes. **A** With different doses of MSCs-EVs (50 μg, 100 μg, 200 μg, 300 μg and 400 μg), qRT-PCR experiment was performed to detect the expression of circHIPK3 post co-culture with chondrocytes. With MSCs-circHIPK3-EVs or a vector (PBS) as the control, **B** EdU experiment was conducted to detect the proliferation after co-culture with chondrocytes. With a vector (PBS) as the control, **C–D** Transwell experiment and cell wound scratch assay were performed for the detection of cell migration. With a vector (PBS) as the control, **E** Flow cytometry was utilized for the detection of cell apoptosis post co-culture with chondrocytes. *P < 0.05, **P < 0.01

Previous reports have shown that circHIPK3 is enriched in EVs of various cells and is important in many biological processes [53]. However, its application in OA remains unknown. Firstly, different doses of MSCs-EVs (50, 100, 200, 300 and 400 μg) were made to co-culture with chondrocytes, then the expression of circHIPK3 was tested using qRT-PCR experiment. The results revealed that the concentration of 200 μg EVs/mL was the minimum concentration to promote the expression of circHIPK3 of chondrocytes after co-culture (Fig. 2A). Therefore, we selected 200 μg EVs/mL MSCs-EVs or MSCs-circHIPK3-EVs for chondrocyte treatment and other in vitro cell experiments. In addition, we also isolated the EVs derived from circHIPK3-overexpressing MSCs (MSCs-circHIPK3-EVs). Then, EdU assay, Transwell assay, and cell wound scratch assay were conducted to verify the effects of MSCs-EVs and MSCs-circHIPK3-EVs on chondrocytes. The results showed that MSCs-EVs and MSCs-circHIPK3-EVs remarkably enhanced the proliferation and migration of chondrocytes, while MSCs-circHIPK3-EVs improved cell proliferation and invasion more effectively than MSCs-EVs (Fig. 2B–D). Additionally, we detected the effect of MSCs-EVs and MSCs-circHIPK3-EVs on chondrocyte apoptosis using flow cytometry. As is shown in Fig. 2E, both MSCs-EVs and MSCs-circHIPK3-EVs could significantly suppress cell apoptosis. Similarly, MSCs-circHIPK3-EVs inhibited chondrocyte apoptosis more effectively than MSCs-EVs. These results demonstrate that MSCs-circHIPK3-EVs might play a vital role in the proliferation and migration of chondrocytes.

### MSCs-circHIPK3-EVs up-regulated COL2A1, Aggrecan, and SOX9 and decreased MMP-13 and Runx2 expression

**Fig. 3:**
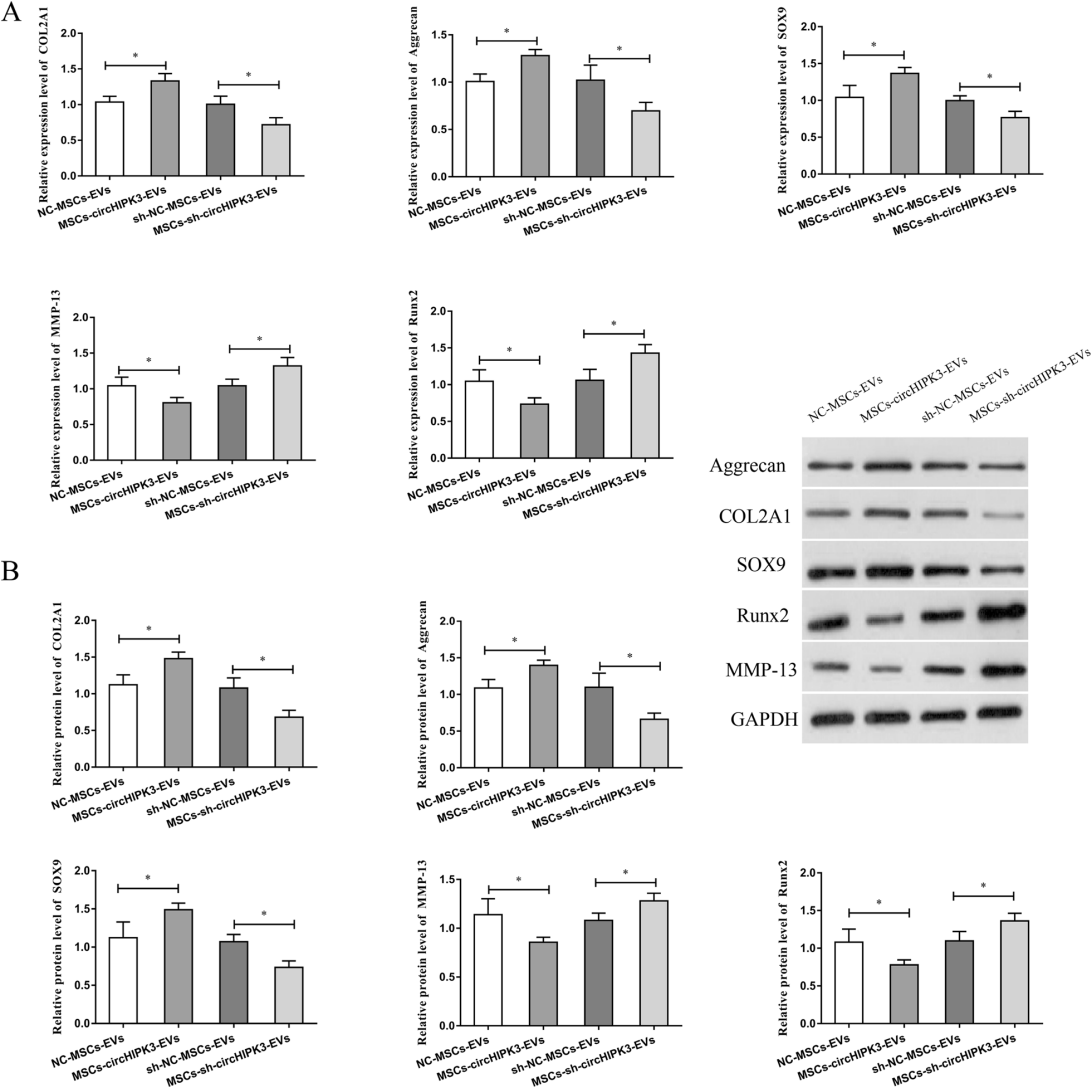
MSCs-circHIPK3-EVs up-regulated COL2A1, Aggrecan, and SOX9 and decreased MMP-13 and Runx2 expression. **A** After the treatment of OA mouse derived chondrocytes with MSCs-circHIPK3-EVs, the expression changes in these key cartilage genes (COL2A1, Sox9, Runx2, Aggrecan and MMP-13) by qRT-PCR. **B** After OA mouse derived chondrocytes were treated with MSCs-circHIPK3-EVs, Western blot was performed to detect the expression change in these key cartilage genes (Sox9, COL2A1, Aggrecan, Runx2 and MMP-13). *P < 0.05

COL2A1 and Aggrecan are important for the normal development and function of cartilage. SOX9 is the target gene of NF-κB, while NF-κB is an important factor involved in OA. Runx2 is a transcription factor involved in chondrocyte differentiation and hypertrophy, and MMP-13 has been implicated in cartilage degradation. Hence, we detected the mRNA and protein expression of these key cartilage genes in OA mouse-derived chondrocytes after treatment with MSCs-circHIPK3-EVs to determine the role of MSCs-circHIPK3-EVs in chondrocytes. The results indicated that, compared with the control group, MSCs-circHIPK3-EVs treatment could lead to obviously higher expression of COL2A1, Aggrecan, and SOX9, and notably lower expression of MMP-13 and Runx2 in chondrocytes (Fig. 3A, B). Besides, we also observed that MSCs-sh-circHIPK3-EVs treatment significantly inhibited the expression of COL2A1, Aggrecan, and SOX9, and promoted the expression of MMP-13 and Runx2 in chondrocytes (Fig. 3A, B). All above results indicate that MSCs-circHIPK3-EVs might inhibit chondrocyte degradation via regulating the expression of key cartilage genes.

### CircHIPK3 promoted MYH9 expression by binding to miR-124-3p

**Fig. 4:**
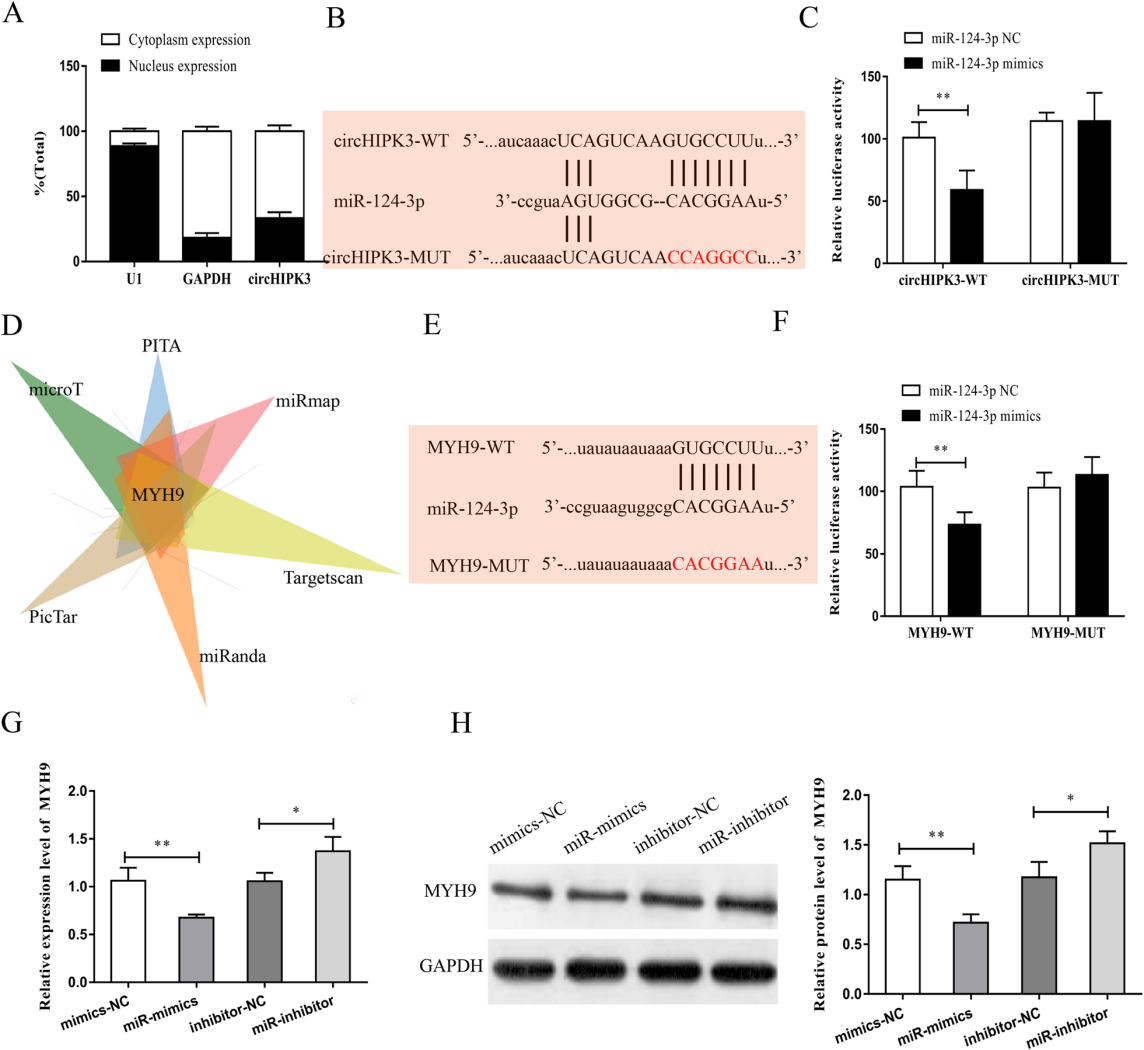
CircHIPK3 promoted MYH9 expression by binding to miR-124-3p. **A** Subcellular localization of circHIPK3 in chondrocytes was detected by nucleoplasmic separation experiment. **B** Binding sites between circHIPK3 and miR-124-3p were predicted by Bioinformatics website, and circHIPK3 wild plasmids (circHIPK3 wt) and circHIPK3 mutant plasmids (circHIPK3 mut) were constructed. **C** The results of dual luciferase reporter gene revealed that miR-124-3p bound to circHIPK3. **D** Bioinformatics website was searched to predict target genes that might bind to miR-124-3p (microT, PITA, PicTar, miRanda, Targetscan and miRmap). **E** Bioinformatics website was searched to predict binding sites of MYH9 to miR-124-3p, and construct MYH9 wild plasmids (MYH9 wt) and circHIPK3 mutant plasmids (MYH9 mut). **F** MiR-124-3p bound to MYH9 based on the dual luciferase reporter gene. **G** MRNA expression of MYH9 was detected by qRT-PCR after upregulation or suppression of miR-124-3p in chondrocytes. **H** Protein expression of MYH9 was detected by Western blot post upregulation or suppress of miR-124-3p in chondrocytes. *P < 0.05, **P < 0.01

Recent studies have shown that circRNA can act as a ceRNA to regulate downstream genes expressionby binding to miRNAs. We first detected the subcellular localization of circHIPK3 using nucleoplasmic separation experiments and observed that circHIPK3 was mainly located in the cytoplasm in chondrocytes (Fig. 4A). This suggested that circHIPK3 might participate in the regulation at the post-transcriptional level. Subsequently, we predicted the miRNAs that might bind to circHIPK3 using the bioinformatics website (Starbase, http://starbase.sysu.edu.cn/) and analyzed their binding ability. We found that several miRNAs were candidate binding targets of circHIPK3. With the highest binding score to circHIPK3 and a documented role in OA [56], miR-124-3p was selected for the follow-up research. Based on the predicted binding sites, we designed and built circHIPK3 wild-type plasmids and mutant-type plasmids (Fig. 4B). The results of dual luciferase reporter gene experiment revealed that miR-124-3p could bind to circHIPK3 (Fig. 4C). Furthermore, we predicted the target genes that might bind to miR-124-3p using bioinformatics softwares (microT, PITA, PicTar, miRanda, Targetscan, and miRmap). With the highest binding score and association with the occurrence and development of OA by selecting the intersection, MYH9 was selected for the subsequent experiments (Fig. 4D). Similarly, MYH9 wild-type plasmids and mutant-type plasmids were designed and constructed using the predicted binding sites (Fig. 4E). The results of the reporter gene analysis showed that miR-124-3p bound to MYH9 (Fig. 4F). Furthermore, we conducted qRT-PCR and western blot assay to verify the effect of miR-124-3p on MYH9. As were shown in Fig. 4G, 4H, the overexpression of miR-124-3p could significantly suppress the mRNA and protein expression levels of MYH9, while miR-124-3p inhibitor exerted the opposite effect.

### EVs-circHIPK3 derived from MSCs attenuated IL-1β-induced chondrocyte injury

**Fig. 5:**
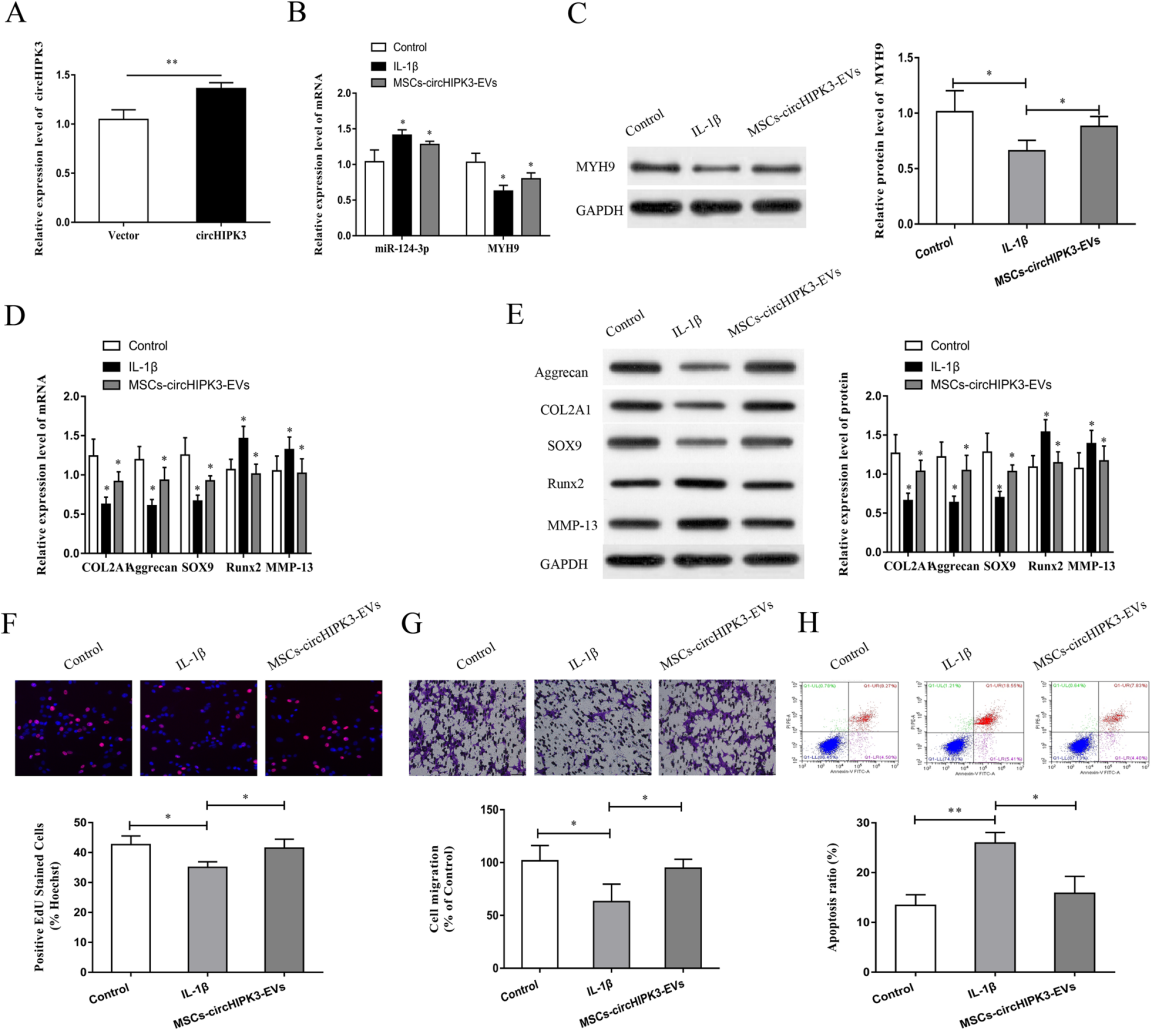
EVs-circHIPK3 derived from MSCs attenuated IL-1β induced chondrocyte injury. **A** Overexpressed circHIPK3 was observed in MSC EVs based on qRT-PCR. **B** With chondrocytes treated with IL-1β and MSCs-circHIPK3-EVs, the expression of miR-124-3p and MYH9 was detected by qRT-PCR. **C** MYH9 expression was detected through Western blot post treating chondrocytes with IL-1β and MSCs-circHIPK3-EVs. **D** With chondrocytes treated with IL-1β and MSCs-circHIPK3-EVs, the expression change in cartilage genes (Aggrecan, COL2A1, Runx2, Sox9 and MMP-13) was detected by qRT-PCR. **E** The expression change in key cartilage genes (Runx2, MMP-13, COL2A1, Aggrecan, and Sox9) was detected through Western blot after chondrocytes were treated with IL-1β and MSCs-circHIPK3-EVs. **F** Cell proliferation was detected through EdU experiment with chondrocytes treated with IL-1β and MSCs-circHIPK3-EVs. **G** Transwell experiment was conducted for the detection of cell proliferation after treating chondrocytes with IL-1β and MSCs-circHIPK3-EVs. **H** A flow cytometer was employed for the detection of cell apoptosis after chondrocytes were treated with IL-1β and MSCs-circHIPK3-EVs. *P < 0.05, **P < 0.01

We further explored whether MSCs-EVs could deliver circHIPK3 and play a role in the treatment of IL-1β-induced chondrocyte injury. We extracted chondrocytes from normal C57BL/6 mice for the study. The chondrocytes were co-cultured with normal culture medium containing 10 ng/ml IL-1β MSCs-circHIPK3-EVs to induce chondrocyte injury. The effectiveness of circHIPK3 overexpression in MSCs-EVs was determined using qRT-PCR (Fig. 5A). The results of qRT-PCR and western blotting showed that the expression of miR-124-3p was increased in IL-1β-induced chondrocytes, while the mRNA and protein expressions of MYH9 were reduced (Fig. 5B, C). Besides, the mRNA and protein expressions of COL2A1, Aggrecan, and SOX9 were reduced and the expressions of MMP-13 and Runx2 were enhanced in the IL-1β-induced chondrocytes (Fig. 5D, E). The results of EdU assay, Transwell assay and flow cytometer indicate that IL-1β treatment considerably suppressed cell proliferation and migration and induced cell apoptosis (Fig. 5F–H). Interestingly, MSCs-circHIPK3-EVs remarkably recovered the IL-1β-mediated negative effect on chondrocytes in vitro (Fig. 5B–H), suggesting that MSCs-circHIPK3-EVs reduced OA chondrocyte injury induced by IL-1β through promoting the proliferation and migration of chondrocytes and suppressing chondrocyte apoptosis. These results verify that MSCs-derived EVs-circHIPK3 could repair IL-1β-induced chondrocyte injury.

### MiR-124-3p overexpression reversed MSCs-circHIPK3-EVs-mediated attenuation of chondrocyte injury

**Fig. 6:**
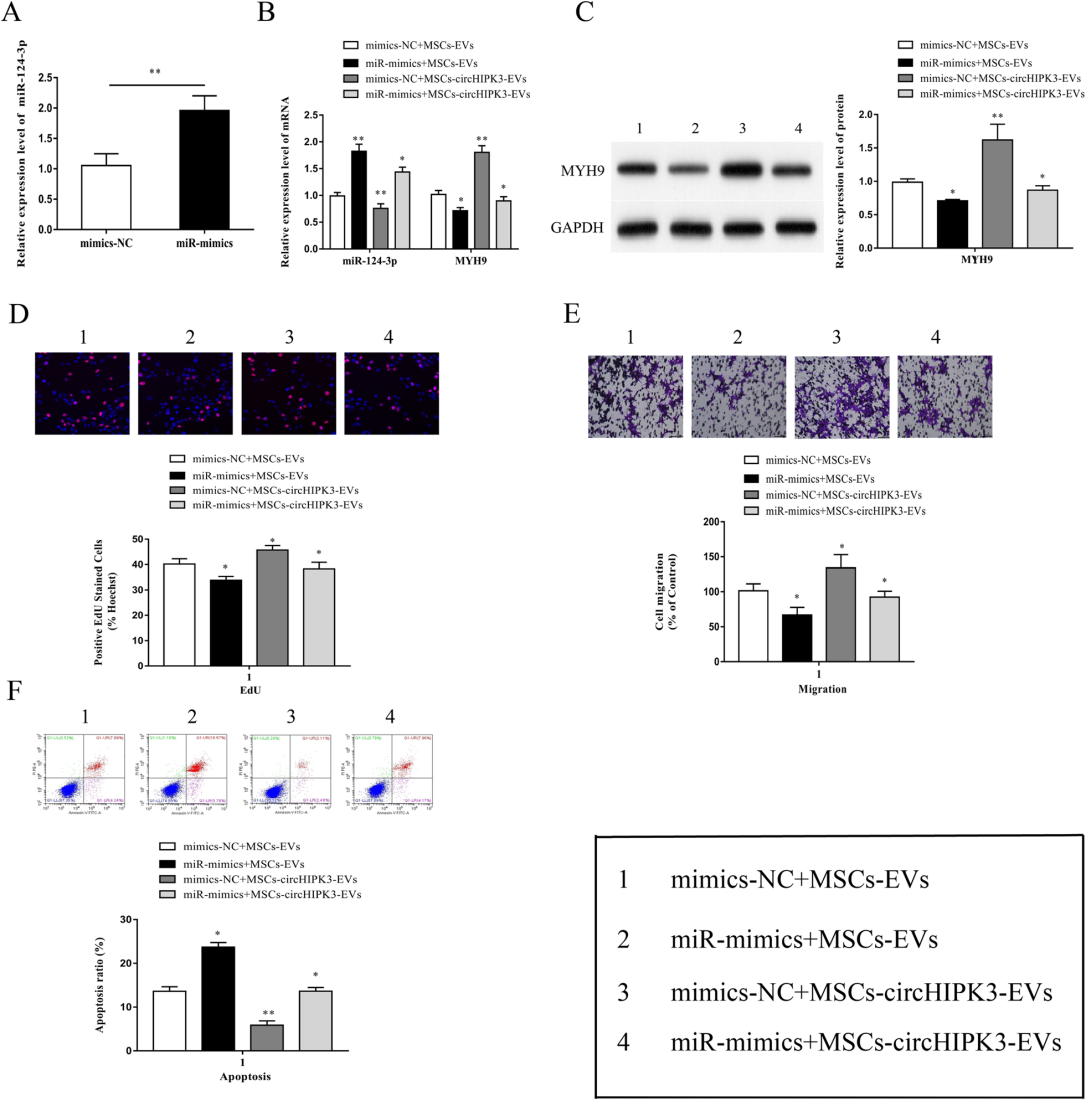
MiR-124-3p overexpression reversed MSCs-circHIPK3-EVs mediated attenuation of chondrocyte injury. **A** The results of qRT-PCR demonstrated that miR-124-3p expression was considerately upregulated in chondrocytes transfected with miR-124-3p mimic. **B–F** MSCs-circHIPK3-EVs was used to co-culture chondrocytes transfected with miR-124-3p mimic, and the chondrocytes were pre-stimulated with IL-1β (10 ng/ml, 24 h). The expression of miR-124-3p and MYH9 was then detected through qRT-PCR (**B**); MYH9 expression was detected via Western blot (**C**); EdU experiment (**D**), Transwell experiment (**E**) and flow cytometry (**F**) were performed for the detection of cell proliferation, migration and apoptosis. *P < 0.05, **P < 0.01

To explore the effect of the circHIPK3/miR-124-3p/MYH9 axis on IL-1β-induced chondrocyte injury, we transfected chondrocytes with miR-124-3p mimics and pre-stimulated chondrocytes with IL-1β. Subsequently, the cells were co-cultured with MSCs-circHIPK3-EVs. The results of qRT-PCR showed that miR-124-3p expression was significantly increased in chondrocytes transfected with miR-124-3p mimics (Fig. 6A). In addition, miR-124-3p mimics significantly reversed the lower MSCs-circHIPK3-EVs-mediated miR-124-3p expression and the higher mRNA and protein expressions of MYH9 (Fig. 6B, C). Meanwhile, the miR-124-3p mimics could remarkedly reversed the MSCs-circHIPK3-EVs promotion of chondrocyte proliferation and migration (Fig. 6D, E), as well as its inhibitory effects on chondrocyte apoptosis (Fig. 6F).

### MYH9 knockdown reversed circHIPK3-EVs-mediated attenuation of chondrocyte injury

**Fig. 7:**
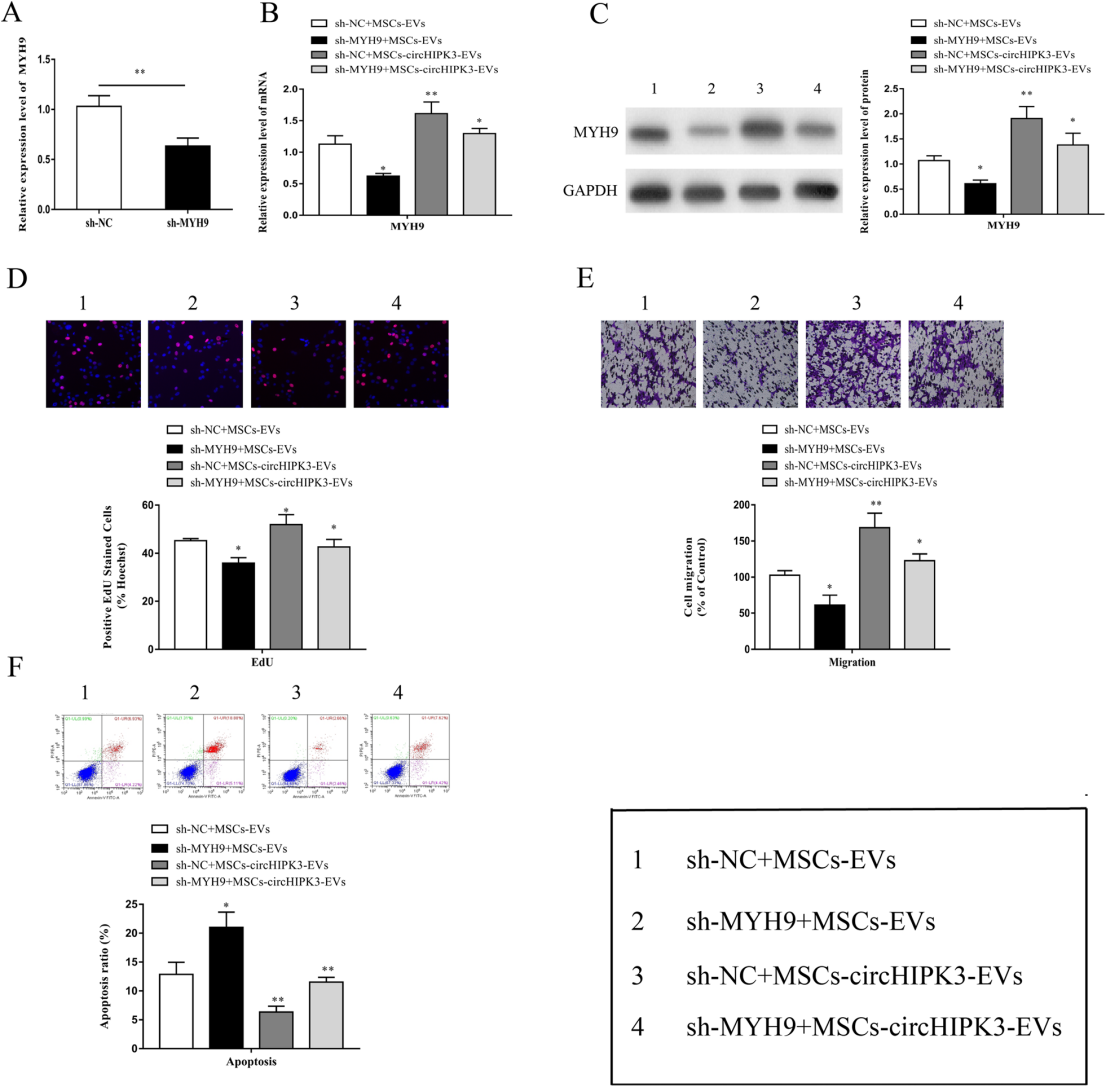
MYH9 knockdown reversed circHIPK3-EVs mediated attenuation of chondrocyte injury. **A** The results of qRT-PCR proved that MYH9 expression was repressed in sh-MYH9 transfected chondrocytes. **B–F** The co-culture was performed by using MSCs-circHIPK3-EVs and sh-MYH9 transfected chondrocytes and the prestimulation was conducted with IL-1β (10 ng/ml, 24 h). MYH9 mRNA expression was then detected by qRT-PCR (**B**); MYH9 protein expression was detected by Western blot (**C**); EdU experiment (**D**), Transwell experiment (**E**) and flow cytometry (**F**) were performed for the detection of cell proliferation, migration and apoptosis. *P < 0.05, **P < 0.01

**Fig. 8:**
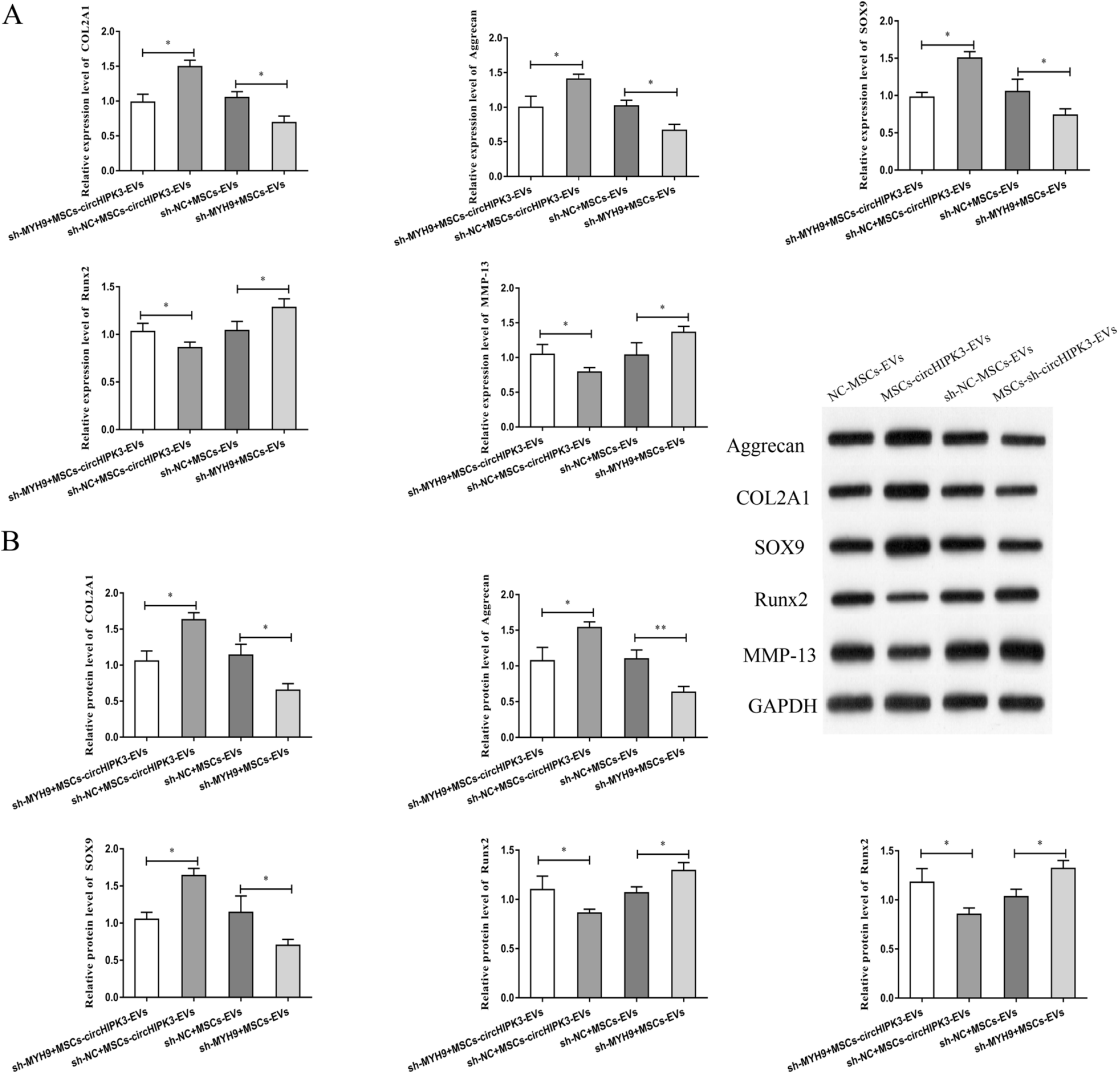
sh-MYH9 decreased COL2A1, Aggrecan and Sox9,  increased MMP-13 and Runx2. **A** After the treatment of OA mouse derived chondrocytes with sh-MYH9, MSCs-EVs and MSCs-circHIPK3-EVs, the expression changes in these key cartilage genes (COL2A1, Sox9, Runx2, Aggrecan and MMP-13) by qRT-PCR. **B** After OA mouse derived chondrocytes were treated with sh-MYH9, MSCs-EVs and MSCs-circHIPK3-EVs, Western blot was performed to detect the expression change in these key cartilage genes (Sox9, COL2A1, Aggrecan, Runx2 and MMP-13). *P < 0.05, **P < 0.01

We transfected chondrocytes with sh-MYH9, and subsequently pre-stimulated the cells with IL-1β. This was followed by co-culture with MSCs-circHIPK3-EVs. The qRT-PCR results indicated that the transfection of chondrocytes with sh-MYH9 obviously suppressed the mRNA expression of MYH9 (Fig. 7A). Moreover, sh-MYH9 could remarkably reverse the increase in mRNA and protein expression of MYH9 mediated by MSCs-circHIPK3-EVs (Fig. 7B, C). In addition, we found that sh-MYH9 notably reversed the MSCs-circHIPK3-EVs promotion of chondrocyte proliferation and migration (Fig. 7D, E) and the suppression of chondrocyte apoptosis (Fig. 7F). Further, we detected the changes in the expression of vital cartilage genes in chondrocytes after sh-MYH9 treatment. The results showed that, in comparison with the control group, the expression of COL2A1, Aggrecan, and SOX9 in chondrocytes was notably reduced and the expression of MMP-13 and Runx2 was significantly enhanced by sh-MYH9 treatment (Fig. 8A, B). These results indicate that MSCs-derived EVs-circHIPK3 might participate in the repair of chondrocyte injury via the miR-124-3p/MYH9 axis.

### MSCs-circHIPK3-EVs inhibited cartilage degradation

**Fig. 9:**
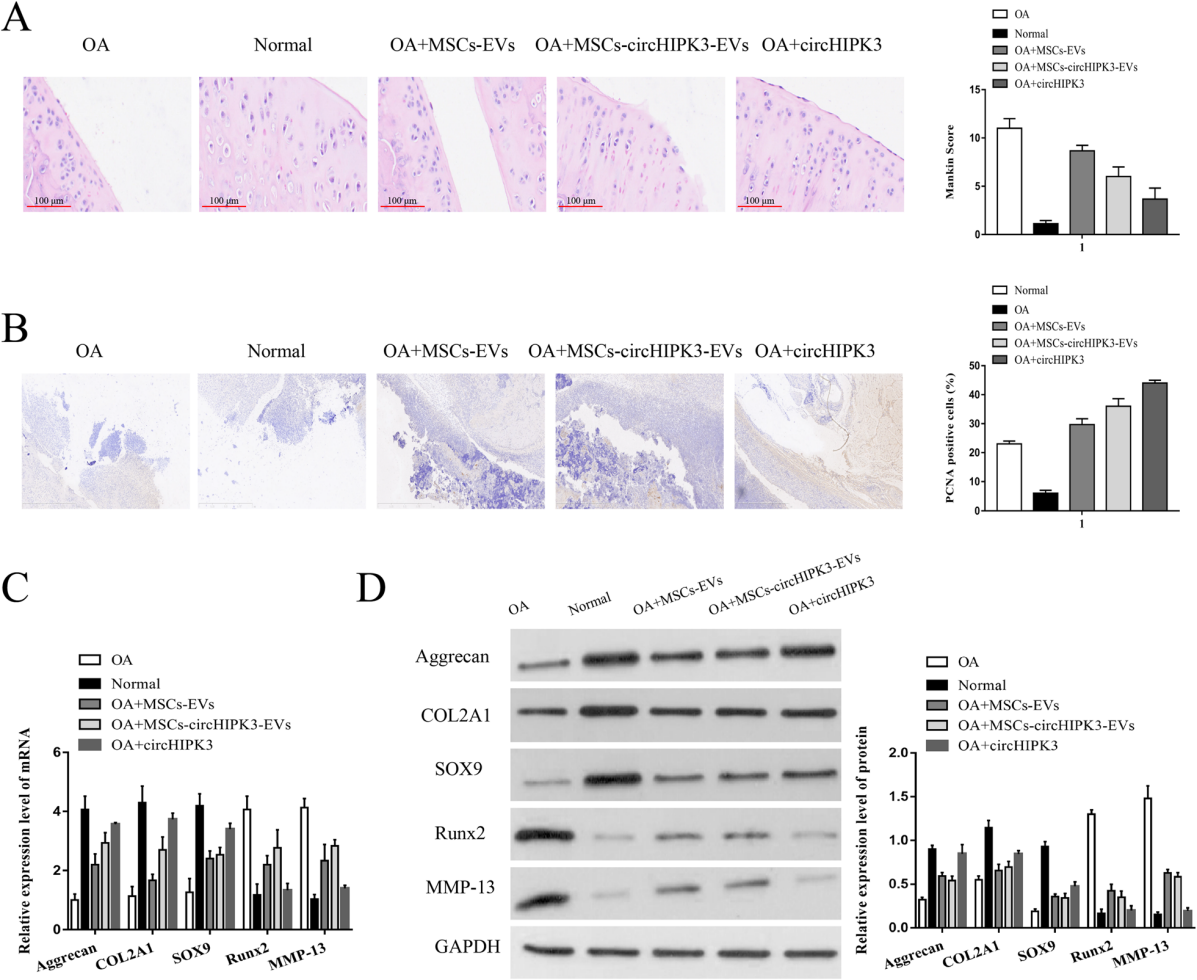
MSCs-circHIPK3-EVs inhibited cartilage degradation. We established the OA model, and divided them into five groups: OA model, Normal, OA + MSCs-EVs, OA + MSCs-circHIPK3-EVs and OA + circHIPK3. **A** HE staining and Mankin score assessment. **B** the number of PCNA-positive cells using immunohistochemistry. **C–D** The mRNA and protein levels of Sox9, COL2A1, Aggrecan, Runx2 and MMP-13 were detected by qRT-PCR and western blot

To verify the protective effect of MSCs-EVs on articular cartilage in vivo, we administered intra-articular injections of MSCs-EVs, MSCs-circHIPK3-EVs, and circHIPK3 into OA models. As shown in Fig. 9A–D, compared with the normal group, the number of chondrocytes and the cartilage thickness were significantly decreased, the Mankin score was significantly increased, the number of PCNA-positive cells was increased, the expression of Aggrecan, COL2A1, and SOX9 was downregulated, meanwhile, the expression of Runx2 and MMP-13 was upregulated in the OA model group. However, compared with the OA group, the MSCs-EVs and MSCs-circHIPK3-EVs groups showed a decreased Mankin score, Runx2, and MMP-13 expression, and an increased PCNA-positive cell, Aggrecan, COL2A1, and SOX9 expression, while a direct injection of circHIPK3 produced a more significant promoting effect. These results suggest that circHIPK3 is present in the MSC-derived EVs and the circHIPK3 EVs can promote cartilage repair.

## Discussion

OA is the most common chronic musculoskeletal disorder and one of the leading causes of disability globally. The pathogenesis of OA is mainly resulted from cartilage damage. MSCs hold great promise for the development of cell-based therapies for various disorders. However, autologous MSCs obtained from bone marrow or adipose tissue have the risk of local complications and infection of the donor. Freitag et al,. used adipose-derived MSCs to treat OA and they found that the treatment group had slight discomforts and bruises after the fat extraction [57]. Ranmuthu et al. also reported adverse events such as local pain and swelling at the fat collection site [58]. Studies from Draganski et al. proved that even if professionally trained personnel performs bone marrow harvesting, there are risks of discomfort, postoperative pain, and infection during the harvesting process, while allogeneic sources may lead to risks such as disease transmission and graft-versus-host disease [59]. EVs are tiny biological vesicles with a bilayer lipid membrane with a diameter of 40–100 nm, originating from the late endosomes of the endocytic system. They are actively released by many types of cells and are widely distributed in saliva, plasma, milk, amniotic fluid, and other body fluids [9, 60]. EVs participate in intercellular information transmission and regulate the recipient cells by binding to specific receptors on the cell surface, directly fusing with the cell membrane of target cells, or been taken up by recipient cells through endocytosis and releasing their contents to the recipient cells [61–63]. The functions of EVs from different cell sources are closely associated with their respective microenvironments. Thus far, the vital roles of MSCs-EVs have been demonstrated in many diseases. MSCs-EVs could restrain chondrocyte apoptosis and promote chondrocyte proliferation [64–66]. In recent years, an increasing number of studies have provided strong evidence that MSCs-EVs could participate in the repair process by transporting functional proteins or RNA. For instances, transforming growth factor beta 1 (TGF-β1)-modified MSCs-derived exosomal miR-135b relieves cartilage injury by promoting the polarization of M2 synovial macrophages via targeting mitogen-activated protein kinase 6 (MAPK6) [38]. MSCs-derived exosomes could relieve OA by modulating the miR-124/NF-kB and miR-143/ROCK1/TLR9 signaling pathways [67]. Besides, exosomal-miR-9-5p, which is secreted by bone marrow-derived MSCs, could alleviate OA by restraining syndecan-1 [68]; human mesenchymal stem cell (hMSC)-derived exosomal-miR-26a-5p alleviates OA by down-regulating PTGS2 [69]. However, the specific mechanism of MSCs-EVs in preventing the development of OA remains to be further explored. Nevertheless, MSC-EVs offer the opportunity to develop a novel therapeutic approach for OA patients.

In this study, we isolated EVs from MSCs and observed that MSCs-EVs could peomote the proliferation and migration of chondrocyte and inhibit cell apoptosis. Furthermore, we found that MSCs-circHIPK3-EVs improved cell proliferation and migration and inhibited cell apoptosis more effectively than MSCs-EVs. MSCs–EVs-circHIPK3 reversed the down-regulation of chondrogenic genes (COL2A1, Aggrecan, and SOX9) and the upregulation of hypertrophy markers (MMP-13 and Runx2) in OA chondrocytes and IL-1β-induced chondrocytes. In addition, MSCs-circHIPK3-EVs could result in a significant promotion of chondrocyte proliferation and migration and the suppression of chondrocyte apoptosis. These findings suggest that MSCs-circHIPK3-EVs hold the promise to be a novel cell-free therapy for treating OA.

It is well known that circRNAs could act as a ceRNA to regulate the expression of downstream target genes by binding to miRNAs [70–72]. After analyzing the binding score and bio-function, we speculated that miR-124-3p might be the candidate binding target. The significant roles of miR-124-3p have been demonstrated in various tumors, such as gastric cancer [73], ovarian cancer [74], and prostate cancer [75]. Meanwhile, Chiu et al*.* observed that gamma-mangostin isolated from *Garcinia mangostana* L. suppresses inflammation and relieves OA symptoms by modulating miR-124-3p/IL-6/NF-kappaB signaling [56]. Wang et al. found that down-regulation of lncRNA SNHG14 inhibits FSTL-1-mediated activation of NLRP3 and TLR4/NF-κB signalling pathway activation by targeting miR-124-3p, thus attenuating inflammatory reactions in OA [76]. Ni et al. demonstrated that Lycium barbarum polysaccharide could protect ATDC5 cells from IL-1β-evoked injury through up-regulating miR-124 via blocking NF-κB and JNK pathways [77]. Therefore, we selected miR-124-3p for the further studies. The results of our studies showed that circHIPK3 could bind to miR-124-3p, and the overexpression of miR-124-3p significantly inhibit chondrocyte proliferation and migration and promote chondrocyte apoptosis. In addition, miR-124-3p could mimics could obviously reduce the expression of COL2A1, Aggrecan, and SOX9, and elevate the expression of Runx2 and MMP-13. These results indicate that circHIPK3 might be involved in the progression of OA by binding to miR-124-3p.

Further predictive functional analysis revealed that MYH9 might be the target of miR-124-3p. MYH9, a class of MYH9 gene encoding “gastric” framework-related protein, is a hexamer composed of two 220 kDa heavy chains, two 17 kDa essential light chains, and two 20 kDa regulatory light chains, widely expressed in cells and tissues [78–80]. MYH9 has been confirmed to have vital regulatory effects in papillary thyroid cancer [81], osteosarcoma [82], gastric cancer [83], melanoma [84] and other diseases. However, the role of MYH9 in OA remains unknown previously. Through the dual luciferase reporter gene experiments and qRT-PCR experiments, we find that miR-124-3p could target MYH9 and suppress the expression of MYH9. Additionally, miR-124-3p overexpression and MYH9 knockdown could reverse the attenuation of IL-1β induced chondrocyte injury mediated by MSCs-circHIPK3-EVs.

There are several limitations of our study. First, we should explore the potential circRNAs secreted by the MSCs-EVs through next- generation sequencing technology with the selected circHIPK3. We will focus on it in the further studies. Second, we will conduct a more in-depth study on the mechanism of circHIPK3 in OA. Third, owing to the experimental conditions, we did not study other sources of MSCs. We will try to explore more in the future studies.

**Fig. 10:**
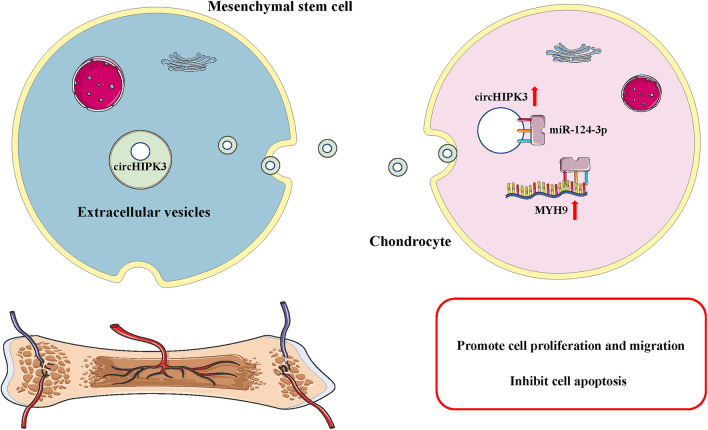
A schematic diagram of MSCs-circHIPK3-EVs in OA

In general, the findings obtained from this study suggest that MSCs-circHIPK3-EVs could promote the proliferation and migration and suppress cell apoptosis of chondrocytes. MSCs-circHIPK3-EVs could prevent the occurrence of OA by increasing the expression of COL2A1, Aggrecan, and SOX9, and down-regulating MMP-13 and Runx2. The results of animal experiments indicate that the MSCs-circHIPK3-EVs could promote cartilage repair. Mechanistically, circHIPK3 could directly bind to miR-124-3p and subsequently elevate the expression of the target gene MYH9 (Fig. 10). Our findings highlight that the future therapeutic strategies for OA should be directed toward the artificial overexpression of circHIPK3, which may potentially be clinically viable targets in the treatment of cartilage damage.

## Conclusions

In summary, MSCs- EVs-circHIPK3 transplantation might promote chondrocyte proliferation and migration and suppress chondrocyte apoptosis via the miR-124-3p/MYH9 axis to promote cartilage repair. Our findings emphasize the possible mechanism of cell delivery of MSCs-EVs-circHIPK3 for the treatment of OA.

## Materials and methods

### Culture and identification of MSCs

MSCs from the American Type Culture Collection (ATCC® PCS-500–012™, ATCC, Manassas, VA, USA) were cultured in Dulbecco’s modified Eagle’s medium (DMEM)/F12 (Gibco) with 10% fetal bovine serum (FBS, Gibco) in a humidified atmosphere with 5% CO_2_ and 95% air at 37 °C. The third passage of MSCs was used in the subsequent experiments. An inverted microscope was used to observe the morphological characteristics of the MSCs. MSCs of the third generation were washed twice with PBS for cell sorting, diluted to 1.0 ~ 2.0 × 10^7^ cells/mL with PBS, and then incubated with phycoerythrin (PE)-conjugated CD11b (1/500, ab213186, Abcam, Cambridge, MA, USA), CD19 (20 µl for a 100 µl sample, ab18229, Abcam, Cambridge, MA, USA), CD34 (1/500, ab223930, Abcam, Cambridge, MA, USA) and allophycocyanin (APC)-conjugated antibody CD73 (5 µl for 10^6^ cells, ab155378, Abcam, Cambridge, MA, USA) and fluorescein isothiocyanate (FITC)-conjugated antibody CD90 (10 µl of the working dilution to label 10^6^ cells in 100 µl, ab11155, Abcam, Cambridge, MA, USA), CD105 (10 µl of the working dilution to label 10^6^ cells in 100 µl, ab11415, Abcam, Cambridge, MA, USA), and HLA-DR (1 µl for 10^6^ cells, ab1182, Abcam, Cambridge, MA, USA) away from light at 4 °C for 40 min. Subsequently, cells were washed with cold PBS, centrifuged at 1,500 g for 5 min, resuspended in PBS, and ultimately performed using a FACScan flow cytometer (BD Biosciences).

### Extraction and identification of MSCs-derived EVs

EVs isolation was carried out using ultracentrifugation [85]. After MSCs grew to 80% confluence, they were rinsed with PBS and then cultured in DMEM containing EVs-free fetal bovine serum for 48 h. EVs were extracted using ultracentrifugation at 3,000 × *g* for 15 min, and the cell culture medium was collected. Medium incubation was performed using 0.5% of the volume of total EVs isolation reagent (Thermo Scientific, Waltham, MA, USA) overnight at 4 °C. The mixture was centrifuged at 12,000 × *g* for 1 h and the supernatant was discarded. The EVs pellets were resuspended in PBS and prepared for identification.

EVs were subjected to morphological observation using TEM. Briefly, we precipitated the prepared exosomes into 50–100 μL of 2% PFA, and added 5 μL of the exosomal suspension to the Formvar-carbon sample copper net. Then we prepared 2–3 copper meshes for each exosomal sample, sealed them, and let the Formvar membrane absorb for 20 min in a dry environment. Moreover, 100 μL PBS was added to the parafilm, keeping the Formvar membrane facing down. The copper mesh was placed on the PBS droplet for washing. Afterward, the copper mesh was immersed in 50 μL of 1% glutaraldehyde droplets for 5 min, and immersed in 100 μL of ddH_2_O for 2 min. Put the copper mesh on a 50 μL uranyl oxalate drop, and then transferred it to the methylcellulose-UA drop for 10 min (operating on ice), put the copper mesh on the stainless steel ring in the air to dry for 5–10 min, and finally put the copper mesh into the electron microscope observation box, and took photoes at 80 kV condition.

Dynamic light scattering was employed to detect the diameter of the EVs. Briefly, Nanosight NS300 analyzer was applied to excite the light wavelength (l = 532 nm). EVs samples were diluted with 0.15 M NaCl to appropriate level of optical signal detection. The particles were illuminated by the laser, with their movement captured for 1 min. The recorded movements were analyzed using Nanosight particle tracking software [Version nanoparticle tracking analysis (NTA) 3.1] to calculate EVs the concentrations and size distribution.

For zeta potential analysis, EVs samples were diluted to a final protein concentration of 25 mg/mL firstly. Then the samples were loaded into disposable zeta cells with gold electrodes and allowed to equilibrate for 15 min at 37 °C. Zeta potential measurements consisted in a set of 15 runs, each one resulting from an automatically defined number of subruns (ranging from 10 to 100) and performed on the Zetasizer Nano ZS (Malvern), at a constant voltage of 40 V.

Besides, western blotting was also used to detect EVs-specific marker proteins CD63 (ab216130; 1/1000, Cambridge, MA, USA), CD9 (ab223052; 1/500, Cambridge, MA, USA), CD81 (ab109201; 1/1000, Cambridge, MA, USA), and GM130 (ab52649; 1/1000 Cambridge, MA, USA), for EVs identification.

### Establishment of the OA model

Adult pathogen-free C57BL/6 mice (9-weeks old; 18–24 g) were supplied by Charles River Laboratories (Beijing, China). The experimental procedures were approved by the Animal Care and Use Committee of China Medical University (No.CMU202187). All mice were raised in a well-ventilated room with free access to water and food, and a 12/12 h light/dark cycle at room temperature (20–25 °C). The mice were numbered according to the weight from light to heavy, and any number was selected from the random number table, and the mice were randomly divided into groups according to the corresponding arrangement of animal number and random number table. To establish an experimental OA mouse model, 0.25 mg/mL type II collagenase solution (Worthington Biochemical, Lakewood, NJ, USA) was dissolved in sterile PBS (pH 7.4), filtered, and injected into the knee joint cavity of C57BL/6 mice under anesthesia. The OA mice were treated with PBS, MSCs-EVs, MSCs-circHIPK3-EVs or circHIPK3, respectively (n = 8/group), while the Normal group (n = 8) used the C57BL/6 mice only.

### Isolation and culture of chondrocytes

Chondrocytes were isolated from the knee joints of the C57BL/6 mice. Briefly, the knee tissues from the right knee of the hind legs of OA mice were cut into small pieces (< 1 mm) and incubated with 0.2% trypsin at 37 °C for 30 min. After removing the trypsin solution, 2 h of tissue treatment followed using 2 mL of 0.2% type II collagenase at 37 °C. After 6 h of digestion, the culture flask was taken out every 2 h, vibrated and observed under an inverted microscope. If many chondrocytes were released, the digestion process was stopped. After washing with PBS three times, the pellets were resuspended with 15% FBS and the chondrocyte culture medium was prepared into the chondrocyte suspension with a density of 1 × 10^9^cells/L. Then took some samples and stained with 0.1% trypan blue to calculate the total number of isolated chondrocytes and the percentage of dead cells. The released cells were cultured in DMEM (Gibco) containing 10% FBS, penicillin (100 U/mL), and streptomycin (100 g/mL). The medium was replaced every two days. The changes of cell growth and morphologies were observed under the inverted microscope every day, and some photos were taken in due course.

### Cell transduction and transfection

With MSCs in good logarithmic growth, 2 × 10^5^ cells were seeded in a 96-well culture plate. When the cell confluence reached 70%, Lipofectamine TM 2000 was used for transfection using shRNAs targeting MYH9, miR-124-3p mimic, and their corresponding controls GenePharma (Shanghai, China), followed by 24 h of conventional culture in a cell incubator for subsequent experimental analysis. For overexpressing or silencing of circHIPK3, overexpressed plasmid or shRNA target circHIPK3 or the negative control was inserted into pLKO.1 vector (Biosettia). 293 T cells (4 × 10^5^ cells/well) were cotransfected with pLKO-circHIPK3 (or pLKO-sh-circHIPK3) or pLKO-NC with psPAX2 and pMD2.G by Lipofectamine 2000 (Invitrogen). 48 h later, lentiviruses were harvested. MSCs were infected with LV-circ or LV-NC with 8 mg/mL polybrene by ViraPower Packaging Mix (ThermoFisher). Stable cell lines were obtained by treatment with 5 μg/mL puromycin (Sigma Aldrich) for 7 days. The sequences for transfection were listed in Table 1.

**Table 1:** Sequences of primers for transfection

Name		Sequence
circHIPK3 shRNA	Sense	5’-GATCCCCTCTCGCTACTACACCTATGCTTCCTGTCACATAG GTGTAGTAGCGAGATTTTTGGAAA-3’
	Anti-sense	5’-AGCTTTTCCAAAAATCTCGCTACTACACCTATGTGACAGG AAGCATAGGTGTAGTAGCGAGAGGG-3’
miR-124-3p mimics	Sense	5’-UAAGGCACGCGGUGAAUGCC-3’
	Anti-sense	5’-CAUUCACCGCGUGCCUUAUU-3’
miR-124-3p NC	Sense	5’-GCACCGTCAAGGCTGAGAAC-3’
	Anti-sense	5’-UUCUCCGAACGUGUCACGUTT-3’
MYH9 shRNA	Sense	5’-CACCGATACTTATCGGCAGCT TGCTGCGAACAGCAAGCTGCCGATAAGTA-3’
	Anti-sense	5’-AAAATACTTATCGGCAGCTT GCTGTTCGCAGCAAGCTGCCGATAAGTATC-3’
MYH9 NC	Sense	5’-CCGGCAACAAGATGAAGAGCAC CAACTCGAGTTGGTGCTCTTCATCTTGTTGTTTTTG-3’
	Anti-sense	5’-AATTCAAAAACAACAAGATGA AGAGCACCAACTCGAGTTGGTGCTCTTCATCTTGTTG-3’

### Luciferase activity assay

CircHIPK3 (or MYH9) wild and mutant dual-luciferase reporter gene plasmids were engineered based on the base sequence by GenePharma (Shanghai, China). After 6 h of transfection using Lipofectamine 2000 co-transfection plasmids and miR-124-3p mimics or miR-124-3p NC, the culture medium was replaced with DMEM containing 10% FBS. According to the manufacturer’s instructions, the Promeg kit (Promega, Madison, WI, USA) was employed in order to measure the fluorescence intensity at 560 nm (firefly relative luciferase units [RLU]) and 465 nm (renilla RLU), and the ratio between firefly RLU and renilla RLU was used to determine the binding strength.

### Cell proliferation assay

Cell proliferation was determined using an EdU kit (RiboBio, China) according to the manufacturer’s instructions. After seeding cells in a 96-well plate at 4,000 cells/well, a 50 mM EdU solution was added to the culture medium. After 24 h, the cells were fixed with 4% formaldehyde, followed by permeabilisation with 0.5% Triton X-100, incubation with EdU reaction mixture, and 100 uL Hoechst33342 counterstaining. A fluorescence microscope was used to record the staining results. Five random fields were selected using a microscope to photograph and calculate cell proliferation.

### Cell migration assay

Transwell inserts (8 μm, Corning, USA) placed in a 24-well plate were used. Then 4 × 10^4^ cells suspended in FBS-free medium were seeded, where 500 μl of medium containing 10% FBS was added in the bottom. Cells were allowed to penetrate for 24 h, and those on the bottom were fixed in methanol for 15 min and visualized by crystal violet for 20 min. Invasive cells were quantified by capturing 5 random fields per well (× 200). For wound healing assay, cells were seeded in 6-well plates. After cells reached 80 − 90% confluence, a 100 μL pipette tip was used to draw a straight wound on the confluent monolayer. The cells were cultured with the serum-free medium and photographed using an inverted microscope 24 and 48 h later.

### Flow cytometry

Cells of each group were harvested into Eppendorf tubes and centrifuged at 1200 rpm for 5 min, and then all the pellet was resuspended via adding 50 μL of binding buffer. Followed by the Annexin V-FITC kit (Beyotime, China) was added in tubes. After the solution was shaken and mixed gently, it was incubated for 15 min at room temperature under the dark environment. Then all the samples were treated with 200 μL of binding buffer and analysed by the FACSCalibur flow cytometer (BD Bioscience, Franklin Lakes, NJ, USA).

### Reverse transcription quantitative polymerase chain reaction (qRT-qPCR)

TRIzol reagent (Invitrogen, Carlsbad, CA) was used to extract the total RNA from chondrocytes or mouse knee cartilage. The Primescript RT kit (Takara, Chiga, Japan) was used for reverse transcription of cDNA according to the manufacturer’s instructions. Reverse transcription-PCR was performed using SYBR Green RT-PCR kit (Takara, Chiga, Japan). The samples were allowed to react at 37 °C for 15 min, 85 °C for 5 s and 4 °C for the remaining time. The cDNA generated following RT was maintained in a -80 °C refrigerator. The reaction conditions of qRT-PCR were set as follows: pre-denaturation at 95 °C for 30 s, with 40 cycles of denaturation at 95 °C for 10 s, annealing at 60 °C for 20 s, and extension at 70 °C for 10 s. GAPDH and U6 were used as the internal references. The primer sequences are listed in Table 2.

**Table 2:** Sequences of primers for qRT-PCR

**Name**		**Sequence**
hsa-circHIPK3	Forward	5’- GTGATCCGGCCTGTTCTTCA -3’
	Reverse	5’- TGACTGGCCGATCCAAAGTC -3’
hsa-miR-124-3p	Forward	5’- ACAGGCTAAGGCTCCCAGTGAA -3’
	Reverse	5’- CGCAGGGTCCGAGGTATTC -3’
hsa-MYH9	Forward	5’- CAGCAAGCTGCCGATAAGTAT -3’
	Reverse	5’- CTTGTCGGAAGGCACCCAT -3’
hsa-GAPDH	Forward	5’-GCACCGTCAAGGCTGAGAAC-3’
	Reverse	5’-TGGTGAAGACGCCAGTGGA-3’
hsa-U6	Forward	5’-CTCGCTTCGGCAGCACA-3’
	Reverse	5’-AACGCTTCACGAATTTGCGT-3’
mmu-COL2A1	Forward	5’- CCCGCCTTCCCATTATTGAC -3’
	Reverse	5’- GGGAGGACGGTTGGGTATCA-3’
mmu-Aggrecan	Forward	5’- ATTTCCACACGCTACACCCTG -3’
	Reverse	5’- TGGATGGGGTATCTGACTGTC -3’
mmu-SOX9	Forward	5’-AGTACCCGCATCTGCACAAC-3’
	Reverse	5’-ACGAAGGGTCTCTTCTCGCT-3’
mmu-RUNX2	Forward	5’-GCACCGTCAAGGCTGAGAAC-3’
	Reverse	5’-GGATCTCGCTCCTGGAAGATG-3’
mmu-MMP-13	Forward	5’- TGTTTGCAGAGCACTACTTGAA-3’
	Reverse	5’- CAGTCACCTCTAAGCCAAAGAAA -3’
mmu-miR-124-3p	Forward	5’- ACAGGCTAAGGCTCCCAGTGAA -3’
	Reverse	5’- CGCAGGGTCCGAGGTATTC -3’
mmu-MYH9	Forward	5’- AGAAGTTGGTATGGGTGCCTT -3’
	Reverse	5’- CCCTGAGTAGTATCGCTCCTTG -3’
mmu-GAPDH	Forward	5’- TGTGTCCGTCGTGGATCTGA -3’
	Reverse	5’- TTGCTGTTGAAGTCGCAGGAG -3’
mmu-U6	Forward	5’- GCTGTGACCCTACAAAGGGA -3’
	Reverse	5’- AGCATCAACTTCAACGCTGC -3’
mmu-circHIPK3	Forward	5’- GCACCTGCAGAGACCTGAAAC -3’
	Reverse	5’- GCAAGTCTCGCCAGTCTCCA -3’

### Western blot

A cell lysis solution (Beyotime, Nantong, China) containing protease inhibitor was added to the chondrocytes or mouse knee cartilage upon protein extraction and placed on ice for centrifugation to collect the supernatant. The protein concentration was determined using a BCA Protein Quantitation Kit (Beyotime, Nantong, China), following the manufacturer’s instructions. The protein lysate was separated by 10% SDS-PAGE protein loading buffer (Beyotime, Nantong, China) and heating to 100 °C. Then transfer to a PVDF (Milipore, USA) membrane began at the end of loading. The antigens were blocked with a 5% skim milk powder blocking solution at room temperature for 2 h and incubated with the specific primary antibody overnight at 4 °C, followed by three times wash in TBST. Then, the membrane was incubated with the corresponding secondary antibody for 2 h at room temperature and then washed in TBST for 3 times (5 min each time). The antibodies against Aggrecan (ab3778; 1 µg/ml), Col2a1(ab239007; 1 µg/ml), Sox9 (ab185966; 1/1000), Runx2 (ab92336; 1/5000), MMP13 (ab231217; 1 µg/ml), MYH9 (ab238131; 1/1000) and GAPDH (ab8245; 1/1000) were purchased from Abcam (Cambridge, MA, USA). HRP-conjugated secondary goat anti-mouse and goat anti-rabbit (Proteintech, USA) was then added dropwise to membrane and incubated at room temperature for 1 h. Then the membrane was washed three tines with TBST. Next, the membrane was immersed in ECL Plus (Millipore, USA) at room temperature for 1 min. The level of protein expression was detected bythe Bio-Imaging System (Bio-Rad, USA). The total protein was determined using GAPDH as an internal reference, with the ratio of gray value of target band to internal reference band was used as relative expression level of protein. The expression level of each protein was detected.

### RNA isolation of nuclear and cytoplasmic fractions

Cells were separated into cytoplasmic and nuclear fractions using a PARIS kit (#AM1921; ThermoFisher). RNA was isolated from each fraction according to the protocol. The RNA levels of circHIPK3, U1, and GAPDH were analyzed using qRT-PCR.

### HE staining and immunohistochemical detection of PCNA

The general conditions of the stained specimens were observed using a light microscope. According to the Mankin scoring system, the severity of cartilage degeneration was evaluated based on pathological changes. After the tissue sections were fixed with 4% paraformaldehyde, they were permeabilised with 0.2% Triton X-100, blocked with 5% BSA, and cultured with PCNA antibody (ab92552, Cambridge, MA, USA) at 4 °C overnight. After washing with PBS three times, the slides were incubated with biotinylated secondary antibody at room temperature for 1 h. Finally, the sections were stained with diaminobenzidine (DAB) and fixed on gelatin-coated glass slides. The average number of PCNA-positive cells was then calculated.

### Statistical analysis

Data are reported as mean ± standard deviation (SD) of the results of, at least, three separate experiments. Student's t-tests and Mann–Whitney U tests were performed to determine the differences between groups, as appropriate. ANOVA and Kruskal–Wallis tests were used for multiple group comparisons. Statistical significance was set at P < 0.05. All statistical analyses were performed using SPSS ver. 20 (IBM Corporation, Armonk, NY, USA).

## Data Availability

The data that support the findings of this study are available from the corresponding author upon reasonable request.

## Abbreviations

EV: Extracellular vesicle
OA: Osteoarthritis
MSCs: Mesenchymal stem cells
CircHIPK3: Circular RNA homeodomain-interacting protein kinase 3
MSCs-EVs: MSCs-Extracellular vesicles
TEM: Transmission electron microscopy
NTA: Nanometer size analysis
miRNA: MicroRNA
MYH9: Myosin heavy chain 9
COL2A1: Collagen type II alpha 1 chain
SOX9: SRY-box transcription factor 9
MMP-13: Matrix metallopeptidase 13
Runx2: RUNX family transcription factor 2
IL-1β: Interleukin1β
ceRNA: Competitive endogenous RNA
ncRNAs: Non-coding RNAs
TGF-β1: Transforming growth factor beta 1
MAPK6: Mitogen-activated protein kinase 6
ROCK1: Rho associated coiled-coil containing protein kinase 1
TLR9: Toll like receptor 9
PTGS2: Prostaglandin-endoperoxide synthase 2
